# The essential *Schizosaccharomyces pombe* Pfh1 DNA helicase promotes fork movement past G-quadruplex motifs to prevent DNA damage

**DOI:** 10.1186/s12915-014-0101-5

**Published:** 2014-12-04

**Authors:** Nasim Sabouri, John A Capra, Virginia A Zakian

**Affiliations:** Department of Medical Biochemistry and Biophysics, Umeå University, Umeå, 901 87 Sweden; Department of Biological Sciences and Biomedical Informatics and Center for Human Genetics Research, Vanderbilt University, Nashville, TN 37235 USA; Department of Molecular Biology, Princeton University, Princeton, NJ 08544 USA

**Keywords:** Pfh1, Pif1 family helicase, G-quadruplex DNA, DNA replication, Schizosaccharomyces pombe, Genome integrity

## Abstract

**Background:**

G-quadruplexes (G4s) are stable non-canonical DNA secondary structures consisting of stacked arrays of four guanines, each held together by Hoogsteen hydrogen bonds. Sequences with the ability to form these structures *in vitro*, G4 motifs, are found throughout bacterial and eukaryotic genomes. The budding yeast Pif1 DNA helicase, as well as several bacterial Pif1 family helicases, unwind G4 structures robustly *in vitro* and suppress G4-induced DNA damage in *S. cerevisiae in vivo*.

**Results:**

We determined the genomic distribution and evolutionary conservation of G4 motifs in four fission yeast species and investigated the relationship between G4 motifs and Pfh1, the sole *S. pombe* Pif1 family helicase. Using chromatin immunoprecipitation combined with deep sequencing, we found that many G4 motifs in the *S. pombe* genome were associated with Pfh1. Cells depleted of Pfh1 had increased fork pausing and DNA damage near G4 motifs, as indicated by high DNA polymerase occupancy and phosphorylated histone H2A, respectively. In general, G4 motifs were underrepresented in genes. However, Pfh1-associated G4 motifs were located on the transcribed strand of highly transcribed genes significantly more often than expected, suggesting that Pfh1 has a function in replication or transcription at these sites.

**Conclusions:**

In the absence of functional Pfh1, unresolved G4 structures cause fork pausing and DNA damage of the sort associated with human tumors.

**Electronic supplementary material:**

The online version of this article (doi:10.1186/s12915-014-0101-5) contains supplementary material, which is available to authorized users.

## Background

DNA helicases are essential for genome stability. They have critical roles in DNA replication, repair and recombination. Multiple human hereditary disorders are linked to mutations in helicase genes. For example, mutations in three of the five human RecQ helicases are associated with increased cancer risk and/or premature aging [1]. A point mutation in the human PIF1 (hPIF1) DNA helicase (hPIF1 L319P) is present in certain families with increased risk of breast cancer and not detected in unaffected controls [2]. This mutation changes a conserved residue within the 21-amino acid Pif1 signature motif that characterizes this family of DNA helicases [3,4].

Pif1 family helicases are found in the genomes of organisms from all three kingdoms [3,4]. Most eukaryotes, including *Schizosaccharomyces pombe* and humans, encode a single Pif1 family helicase, while *Saccharomyces cerevisiae* encodes two, ScPif1 and ScRrm3. Pfh1, the *S. pombe* Pif1 helicase, is essential for maintenance of both the nuclear and mitochondrial genomes [5]. In nuclear DNA, it facilitates replication fork progression through many sites that impede fork progression, such as highly transcribed RNA polymerase II and III genes, replication fork barriers within both the ribosomal DNA (rDNA) and the mating-type locus, and converged replication forks [6,7]. At the mating type locus and rDNA, Pfh1 helps forks move past stable protein complexes. In the absence of Pfh1, double strand breaks (DSBs) occur specifically at these natural fork impediments [6].

Pfh1’s role at a different class of hard to replicate sites, stable non-canonical DNA secondary structures such as the G-quadruplex (G4), has not been systematically explored. G4 structures are stable DNA secondary structures held together by multiple stacked guanine quartets [8]. G4 structures can form within a single DNA molecule (intra-strand) or between different DNA molecules (inter-strand). In virtually all genomes examined so far, DNA sequences that are capable of forming intra-strand G4 structures *in vitro* (G4 motifs) are observed. G4 motifs are highly enriched in G-rich telomeric DNA, where they affect telomerase action and end protection [9,10]. In addition to telomeres, there are more than 300,000 sites in the human genome with the potential to form G4 DNA [11,12], and G4 structures can be detected in human cultured cells with G4 specific antibodies [13,14]. Moreover, G4 structures were more frequent in FANCJ helicase-deficient human cultured cells [14]. In bacteria, budding yeast, and humans, G4 motifs are common in rDNA and promoter regions [15-17].

Although G4 motifs are not frequent sites of DNA damage in wild type (WT) *S. cerevisiae*, in *pif1* mutant cells, replication forks slow and often break at these sites [18]. In WT cells, ScPif1 binds a subset of G4 motifs, and this subset is more likely to be associated with fork slowing and DNA breakage in its absence. Moreover, in cells lacking ScPif1, G4 motifs induce gross-chromosomal rearrangements (GCRs) [19,20]. Although G4 motifs do not induce genome instability in *rrm3* cells, G4-induced GCR events are particularly elevated in *pif1 rrm3* cells, suggesting that ScRrm3 acts as a backup for ScPif1 in suppressing G4-induced DNA damage. Consistent with a role for ScPif1 at G4 motifs *in vivo*, ScPif1 and four of four tested bacterial Pif1 helicases are particularly robust unwinders of G4 structures, even under single-cycle conditions [19]. In human cells, some of the binding sites of the G4 stabilizing agent pyridostatin, co-localize with the binding of hPIF1, suggesting a role of hPIF1 in resolving G4 DNA [21].

Here, we investigated the relationship between G4 motifs and the *S. pombe* Pfh1 helicase. Compared to *S. cerevisiae*, *S. pombe* has a chromosome structure more similar to that of higher eukaryotes and, like human cells, encodes a single Pif1 family helicase. Thus, it is a good model for understanding the functions of hPIF1. We used computational methods to map intra-strand G4 motifs within the genome of *S. pombe* and three other sequenced *Schizosaccaromyces* yeasts to determine their association with genomic features. G4 motifs were significantly enriched in rDNA, telomeres, meiotic DSB hot spots, gene promoters, nucleosome-depleted regions (NDRs), untranslated regions (UTRs), and dubious open reading frames (ORFs), but depleted in ORFs. Using chromatin immunoprecipitation in combination with deep sequencing (ChIP-seq), we found that Pfh1 was bound near approximately 20% of the G4 motifs in the assembled *S. pombe* nuclear genome and that fork slowing and DNA damage, as indicated by association with Cdc20, the leading strand DNA polymerase, and phosphorylated H2A (γ-H2A), respectively, were associated with G4 motifs in Pfh1-depleted cells. Together, our data suggest that Pfh1 is needed to unwind G4 structures; when this unwinding does not occur, forks slow and often break. This increased genome instability in Pfh1-depleted cells could explain the association of hPIF1 mutations with cancer.

## Results

### Identifying G4 motifs in fission yeasts

**Figure 1:**
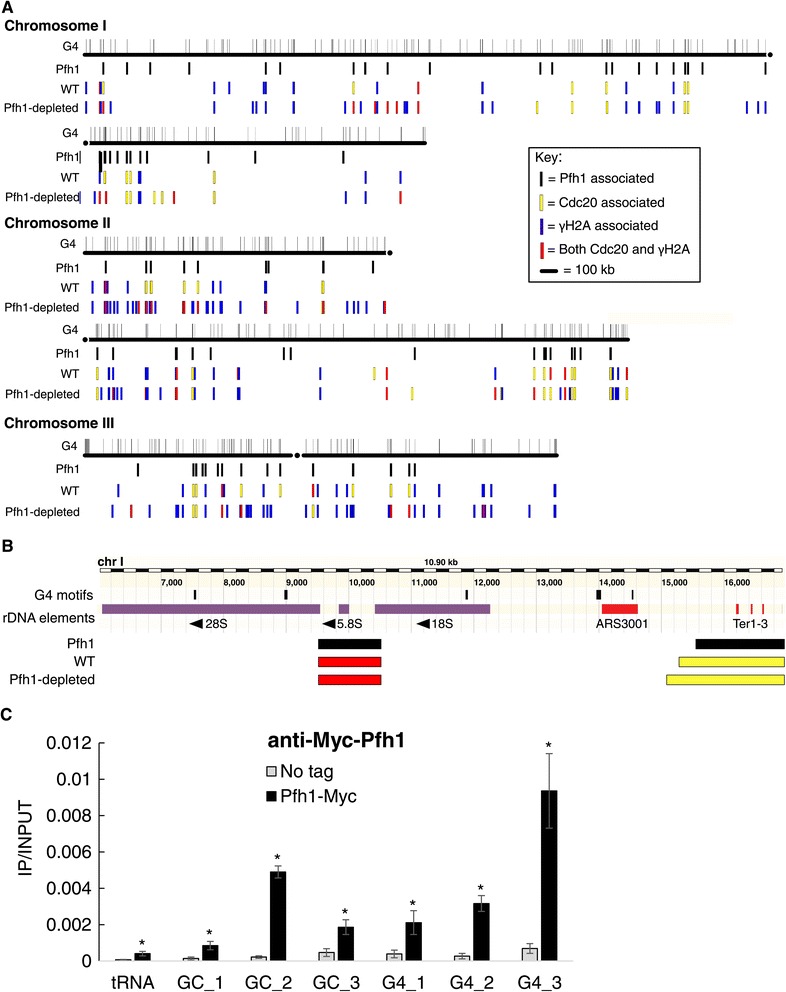
**Distribution of G4 motifs and sites of high DNA polymerase and DNA damage across the** ***S. pombe*** **genome. (A)** Gray lines above each chromosome indicate the locations of the 446 G4 motifs in the assembled *S. pombe* genome. Three data tracks are shown below each chromosome: (1) G4 motifs associated with high Pfh1 occupancy (Pfh1, black); (2) G4 motifs associated with high Cdc20 (yellow), γ-H2A (blue) or both (red) in WT cells; and (3) same information as in (2) but for Pfh1-depleted cells. Note that the genome assembly does not include telomeric repeats and has only three complete copies of the approximately 300 rDNA repeats that are located near both telomeres on chromosome III. **(B)** The location of G4 motifs within a representative rDNA repeat from the left arm of chromosome III (III: 6027–16927). Each of the five G4 motifs (top black bars scaled by G4 motif length) in the 10.9 kb rDNA repeat are on the non-transcribed strand. The arrows denote direction of transcription of the rDNA genes. The locations of Pfh1, Cdc20 and γ-H2A sites are shown below the rDNA annotation track using the same color scheme as in (A). Ter1-3 are natural replication fork barriers, and ARS3001 is a replication origin. **(C)** Pfh1-associated peaks were validated by ChIP-qPCR using an anti-Myc antibody. Association was calculated as immunoprecipitated DNA divided by input DNA (IP/input). Data present the average of three independent biological replicates and error bars are standard deviations. Three GC-rich sites, three G4 motifs and one tRNA gene (tRNA glu.05) were tested. The tRNA gene was used as a positive control for Pfh1 association. All tested sites were significantly bound by Pfh1 compared to the untagged control strain by two-tailed student t-test *P* ≤0.01. ChIP, chromatin immunoprecipitation; qPCR, quantitative PCR.

We performed a genome-wide search for DNA sequences with the potential to form G4 structures on the genomes of the four available *Schizosaccaromyces* species [22]. We identified all sequences that contain four runs of three or more guanine base pairs (bp), ‘G-islands’, separated by ‘loop’ regions of no more than 25 bp (G_≥3_ N_1–25_)_3_ G_≥3_ [15]. Hereafter, sequences matching this pattern are called ‘G4 motifs’. Regions with more than four G-islands separated by ≤25 bp were counted as a single G4 motif. Excluding repetitive DNA (see below), the *S. pombe* genome contained 446 G4 motifs that match this query pattern with a density of 0.036 G4 motifs/kilobase (kb) (Figure 1A; Table 1). The density of G4 motifs was similar across all three *S. pombe* chromosomes (Figure 1A). The *S. octosporus* and *S. cryophilus* genomes contained a similar number and density of G4 motifs as *S. pombe* (Table 1; Additional file 1). The more distantly related *S. japonicus* had roughly four times the number (1,757) and density (0.16 G4 motifs/kb) of G4 motifs as the other fission yeast species, most likely due to the higher GC content of its genome (44% versus 36% in *S. pombe*).

**Table 1:** **The number, density and evolutionary conservation of G4 motifs in the nuclear and mitochondrial (mt) genomes of four fission yeasts**

**DNA sequence**	**Genome size**	**Genome GC fraction**	**Number of G4 motifs**	**G4/bp**	**Fraction of genome (bp) aligned to Sp**	**Number of** ***S. pombe*** **G4 motifs conserved**
*S. pombe* nuclear	12.6 Mb	0.36	446	3.55E-05	1	NA
*S. octosporus* nuclear	11.3 Mb	0.38	539	4.78E-05	0.576	27
*S. cryophilus* nuclear	11.5 Mb	0.38	443	3.85E-05	0.576	20
*S. japonicus* nuclear	11.1 Mb	0.44	1758	1.58E-04	0.435	31
*S. pombe* mt	19.4 kb	0.30	0	0	NA	NA
*S. octosporus* mt	44.2 kb	0.24	0	0	NA	NA
*S. cryophilus* mt	32.7 kb	0.30	1	3.06E-05	NA	NA
*S. japonicus* mt	80.1 kb	0.20	5	6.25E-05	NA	NA

The above estimates for the number of G4 motifs do not include G4 motifs within repetitive telomeric DNA and most of the rDNA. Telomeric DNA was not included in this analysis because it is not in the *S. pombe* genome assembly. However, analysis of *S. pombe* telomeric DNA in a library containing 18 kb of sequenced telomeric DNA (Webb and Zakian, submitted) revealed that it had two orders of magnitude more G4 motifs per kb (4.5 G4 motifs/kb) than bulk nuclear DNA [see Additional file 2]. The presence of G4 motifs in telomeric DNA is not surprising as the *S. pombe* telomeric sequence, G_2-8_TTAC, is GC-rich [23]. However, the density is nearly three times higher than expected in random sequences of the same GC content (1.7 G4 motifs per kb). Assuming 300 bps of telomeric DNA at each of the six chromosome ends, we estimate 1 to 2 G4 motifs per telomere or approximately 10 telomeric G4 motifs per haploid genome.

The *S. pombe* genome assembly contains only three full rDNA repeats, a small fraction of the estimated approximately 300 rDNA repeats in nuclear DNA [24]. Each of the three copies of the 10.9 kb rDNA repeat had five G4 motifs (Figure 1B). Assuming that these G4 motifs are present in all of the rDNA repeats, there are approximately 1,500 G4 motifs in *S. pombe* rDNA, accounting for almost 80% of the G4 motifs in nuclear DNA. The density of G4 motifs in the rDNA (0.45 G4/kb) was significantly greater than expected from random sequences of the same GC content (0.08 G4/kb; *P* = 0.003) and more than ten times higher in rDNA than the average in the nuclear genome (0.45 G4/kb versus 0.036 G4/kb), even though the GC content of the two is similar (38% versus 36% GC content in rDNA versus nuclear genome). Each of the five rDNA G4 motifs was on the non-transcribed strand (Figure 1B): three were in transcribed regions, two in the 28S and one near the start of the 18S rRNA. Of the three G4 motifs in transcribed regions, the one in 18S and one of the two in 28S were conserved in sequence and position between *S. pombe* and *S. japonicus* [25] (sequence data for the rDNA in the other *Schizosaccaromyces* species are not available). The high density of G4 motifs is also true for *S. cerevisiae* [26] and human rDNA [27]. In both *S. pombe* and *S. cerevisiae*, G4 motifs are found only on the non-transcribed strand [15], suggesting a potential role for G4 structures in the rRNA. However, the most highly conserved G4 motif, which is in the 18S rRNA, does not form a G4 structure in an existing crystal structure of the *S. cerevisiae* ribosome [28].

We also determined the G4 motif content of the mitochondrial (mt) DNA of the four fission yeast species, which range in size from 19.4 kb in *S. pombe* to 80.1 kb in *S. japonicus* (Table 1). No G4 motifs were present in *S. pombe* or *S. octosporus* mtDNA, while *S. cryophilus* and *S. japonicus* contained one and five motifs, respectively. These results are in sharp contrast to the very high density of G4 motifs in the AT-rich *S. cerevisiae* mtDNA compared to its nuclear genome (0.37 G4 motifs/kb mtDNA versus 0.055 motifs/kb nuclear DNA) [15]. Hereafter, we will address the events that occur in the 446 G4 motifs found in the published *S. pombe* genome assembly.

### Evolutionary conservation of G4 motifs across fission yeasts

There was little evolutionary conservation of G4 motif locations among the four fission yeast species. In each pairwise combination, only a small number of *S. pombe* G4 motifs (20 to 31) overlapped a G4 motif in the aligned homologous location in another species (Table 1). Moreover, different motifs were maintained between different pairs of species. Excluding the rDNA repeats, only five motifs were conserved between *S. pombe*, *S. octosporus* and *S. cryophilius*, and only one location, the promoter of *cdc13*^*+*^, had a G4 motif in all four species.

This low level of evolutionary conservation is not surprising due to the evolutionary divergence of the available fission yeast genomes. *S. octosporus*, *S. cryophilus* and *S. pombe* diverged from their last common ancestor more than 100 million years ago (mya), while *S. japonicus* diverged more than 200 mya [22]. Studies of the evolutionary turnover of G4 motifs [15] and other regulatory elements [29-31] in yeasts find that most regulatory elements are not conserved over these timescales.

### The genomic distribution of G4 motifs

To investigate potential functions for *S. pombe* G4 motifs, we analyzed the distribution of the G4 motifs with respect to multiple genomic features, such as highly expressed genes, NDRs, meiotic DSB hotspots and so on. (See the Methods for the full list). For these analyses, we first computed the number of overlaps between the G4 motifs and a given feature of interest. Then, to evaluate if the observed association was more or less than expected by chance, we created 1,000 sets of ‘control’ regions. Each of these 1,000 control sets contained 446 random genomic regions—one for each of the actual G4 motifs. Each of the 446 regions in a single control set matched the chromosome, length and GC content of a different observed G4 motif, so the average region length and GC content for each of the 1,000 sets matched that of the actual G4 motifs. Then, for each of these 1,000 sets of control regions, we computed the number of overlaps with the feature of interest. By comparing the number of observed G4 motif overlaps to the 1,000 overlap counts from the control sets, we obtained an empirical estimate of the likelihood of the observed association by chance (that is, a *P*-value). To account for the testing of multiple hypotheses, for each enrichment test, we report q-values, which are the false discovery rate (FDR) analogue of *P*-values and correspond to the FDR if a particular test is called significant [32]. See Methods for more information.

Using this approach, we found that G4 motifs were significantly associated with several genomic features (Table 2; Additional file 3). G4 motifs were more likely to occur in the promoters of RNA polymerase II-transcribed genes (q <0.003), NDRs (q <0.003), meiotic DSB hot spots (q <0.003), 3′ and 5′ UTRs (q = 0.013 and q = 0.003), and within dubious genes (q <0.003) than expected by chance. In contrast, G4 motifs were significantly depleted from ORFs of protein-coding genes (q <0.004), including essential and highly transcribed genes. However, when G4 motifs were found within ORFs, they were significantly more likely to occur on the transcribed strand than expected by chance (219/303, 72%; *P* <1E-12, binomial test). In these cases, the G4 motif would not be present in the mRNA. Thus, any function of these G4 motifs would likely be carried out in DNA. G4 motifs were not significantly associated with long terminal repeats (LTRs), tRNA genes, 5S rRNA genes, origins of replication or centromeres (q >0.05 for all; Additional file 3). Similar association patterns were found when decreasing the loop length from 25 bp to 12 bp (data not shown).

**Table 2:** **Distribution of G4 motifs relative to functional elements in** ***S. pombe***

**Genomic feature**	**Total number of features**	**G4 overlap**	**Expected overlap**	**G4 motif status**	**FDR adjusted q-value**
Promoters	3,237	45	27	Enriched	<0.003
Dubious ORFs	71	13	4	Enriched	<0.003
3′ UTRs	5,144	48	34	Enriched	0.013
5′ UTRs	5,144	47	27	Enriched	0.003
Nucleosome-depleted regions	2,300	54	18	Enriched	<0.003
Meiotic DSB hot spots	288	112	62	Enriched	<0.003
Protein-coding ORFs	5,144	303	382	Depleted	<0.003

**Figure 2:**
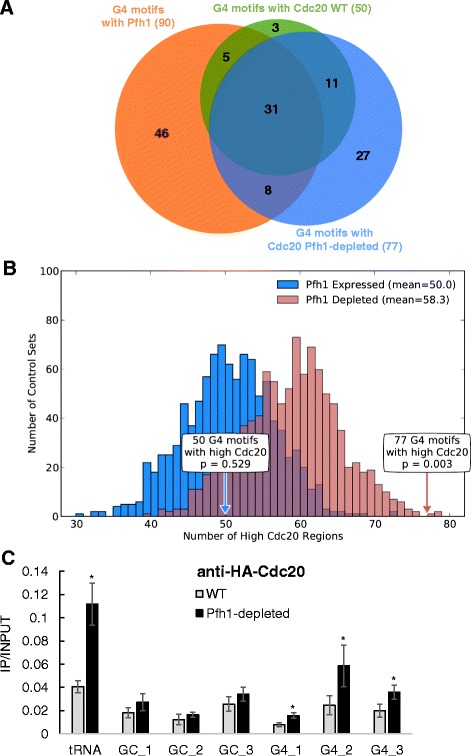
**A subset of G4 motifs have high Cdc20 binding indicative of replication fork pausing in Pfh1-depleted cells. (A)** The Venn diagram gives the overlaps between G4 motifs associated with high Pfh1 (orange circle), high Cdc20 in WT cells (green circle) and high Cdc20 in Pfh1-depleted cells (blue circle). For the most part, G4 motifs with high Cdc20 in WT are a subset of those with high Cdc20 in Pfh1-depleted cells. **(B)** To compare the overall Cdc20 occupancy at G4 motifs in Pfh1-expressing (blue) and Pfh1-depleted cells (red), we estimated the expected distribution of high Cdc20 sites for 1,000 control region sets matched to the observed G4 motifs (histograms). Because the coverage of the genome by Cdc20 is higher in Pfh1-depleted cells, the expected G4 motif association is somewhat higher for Pfh1-depleted cells (58.3; red) than Pfh1-expressing cells (50.0; blue). In Pfh1-expressing cells the 50 G4 motifs with high Cdc20 occupancy (blue arrow) was not different from the expected number (*P* = 0.529). In Pfh1-depleted cells, significantly more G4 motifs were associated with high Cdc20 regions than expected (red arrow; 77 regions; *P* = 0.003). **(C)** Cdc20-3HA peaks were validated in WT and Pfh1-depleted cells by ChIP-qPCR using an anti-HA antibody as described for Figure 1C. In Pfh1-depleted cells, all G4 motifs and the tRNA gene had a significant increase of Cdc20 levels compared to WT cells (two-tailed student t-test, *P* ≤ 0.04). ChIP, chromatin immunoprecipitation; G4, G-quadruplex; qPCR, quantitative PCR; WT, wild type.

**Figure 3:**
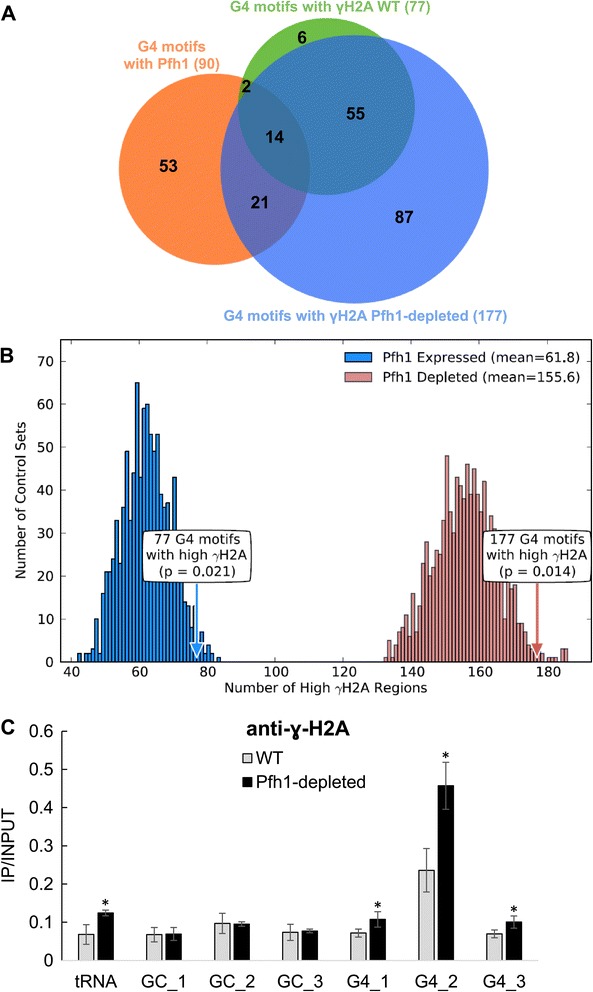
**G4 motifs are significantly associated with DNA damage. (A)** The Venn diagram shows the overlaps between G4 motifs that were associated with high Pfh1 (orange), high γ-H2A in WT cells (green circle), and high γ-H2A in Pfh1-depleted cells (blue circle). Nearly all G4 motifs with high γ-H2A in WT also had high γ-H2A in Pfh1-depleted cells. **(B)** G4 motifs were significantly associated with high γ-H2A in both Pfh1-expressing (blue) and Pfh1-depleted (red) cells. The significance of these associations was determined by comparison to the number of overlaps in 1,000 random control sets in both Pfh1-expressing (blue histogram) and Pfh1-depleted cells (red histogram). When Pfh1 was depleted, there was a dramatic increase in the amount of DNA damage at G4 motifs, as inferred from γ-H2A associations for Pfh1-depleted (155.6; red) versus Pfh1-expressing (61.8; blue) cells. In both contexts, the association of G4 motifs with high γ-H2A was significant (blue arrow; Pfh1-expressing, *P* = 0.021; red arrow; Pfh1-depleted, *P* = 0.014). **(C)** γ-H2A associated peaks were validated in WT and Pfh1-depleted cells by ChIP-qPCR using an anti- γ-H2A antibody as described in Figure 1C except that the primer pairs were located further from each test site (within 5 kb) to detect regions with the highest γ-H2A content. By two-tailed student t-test, all G4 motifs and the tRNA gene had significantly increased γ-H2A in Pfh1-depleted cells compared to WT cells (*P* ≤ 0.05). ChIP, chromatin immunoprecipitation; G4, G-quadruplex; qPCR, quantitative PCR; WT, wild type.

G4 motifs are enriched in promoters of Pol II transcribed ORFs, NDRs, dubious ORFs, UTR and meiotic DSB hot spots. G4 motifs are depleted from ORFs overall, as well as essential and highly transcribed genes. G4 motifs are also enriched in Cdc20 occupied sites in Pfh1-depleted cells (Figure 2), γH2A sites (Figure 3), telomeric repeats and rDNA repeats (Figure 1B).

### Many G4 motifs are Pfh1 associated

Pfh1 is a replisome component that moves with the leading strand polymerase ɛ (Sabouri *et al.*, in preparation). Thus, we anticipated that if some (or all) G4 motifs slow DNA replication, even in wild type cells, they would have higher binding by both Pfh1 and Cdc20, the catalytic subunit of DNA polymerase ɛ. If Pfh1 promotes replication fork progression past G4 motifs, the Cdc20 association at these sites should be even higher in Pfh1-depleted cells. To test these hypotheses, we used a *S. pombe* strain expressing epitope-tagged Pfh1, isolated Pfh1-associated DNA by immunoprecipitation (ChIP), and then sequenced the associated DNA (ChIP-seq). The input DNA for the ChIP was sequenced as a control. Sites with significant Pfh1 occupancy were identified with the Model-based Analysis of ChIP-seq peak calling software (MACS; Zhang *et al*. [49]), using a stringent cutoff for both ChIP and input DNA (*P* <10^-5^). The same strategy was used in all of the ChIP-seq analyses in this paper (Methods section). With these methods, we identified 621 high confidence Pfh1-associated sites in DNA from asynchronously growing cells. Two of these peaks mapped to the rDNA, although not to the G4 motifs in the rDNA (Figure 1B). The assembled *S. pombe* genome lacks telomeric DNA, so this analysis did not assess Pfh1 association with telomeres. However, ChIP-qPCR shows that telomeres were also Pfh1 associated [33].

Of the 621 Pfh1 peaks, 76 (12%) were ≤300 bp, the shearing size of the ChIP DNA, from a G4 motif. Several Pfh1 peaks were associated with more than one G4 motif, so in total, 90 (20%) of the 446 G4 motifs in the assembled nuclear genome were Pfh1 associated (Figure 1A). The observed association between Pfh1 and G4 motifs was significantly greater than expected by chance (*P* = 0.002). However, it was significantly lower than expected when taking the GC-content of the G4 motifs into account using our control region sets (*P* = 0.016). We also validated the ChIP-seq peaks by quantitative PCR (ChIP-qPCR). We compared the association of Pfh1-13Myc or an untagged otherwise isogenic control strain to a tRNA gene (tRNA glu.05), a previously known Pfh1-binding site [6], three GC-rich sites and three G4 motifs (see Methods for details). Pfh1 was significantly associated with all these sites compared to the control strain (Figure 1C). Together, these results suggest that Pfh1 is present not only at many G4 motifs but also at many other sites, especially at other GC-rich sequences, consistent with its being a multi-functional DNA helicase [6]. This finding is also consistent with the behavior of ScPif1, which binds preferentially to G-rich regions, even those unable to form G4 structures, *in vivo* and *in vitro* [19].

### Replication forks pause near G4 motifs in Pfh1-depleted cells

To monitor fork progression at G4 motifs in the presence and absence of Pfh1, we epitope tagged Cdc20 and performed ChIP-seq in WT and Pfh1-depleted cells. Although all sites in the genome are Cdc20-associated at their time of replication, sites where replication forks move slowly are expected to have elevated Cdc20 binding, as seen with DNA Pol2, the catalytic subunit of DNA polymerase ε in *S. cerevisiae* [18,34]. To deplete cells of Pfh1, an essential protein, we used the thiamine repressible nmt81 promoter [5]. Growth of nmt-Pfh1-GFP cells in thiamine for 12 hours reduces Pfh1 expression, so that Pfh1 is no longer detected by western blot analysis [5,6]. Hereafter, cells treated in this way are referred to as ‘Pfh1-depleted cells’ and untreated cells as ‘WT’/Pfh1-expressing’ cells.

Using these methods, we identified 485 sites of high Cdc20 binding in WT cells, including 50 G4 motifs (Figures 1A and 2A). This number of G4 motifs was not significantly different from the expected association based on the 1,000 control sets (*P* = 0.529; Figure 2B, blue arrow). Thus, in WT cells, G4 motifs were not enriched among sites of replication fork pausing compared to other GC-rich regions.

There were 517 high confidence Cdc20 peaks in Pfh1-depleted cells, modestly more than the number in WT cells. However, in contrast to WT cells, G4 motifs were highly enriched among the high Cdc20 binding sites in Pfh1-depleted cells (77 of the G4 motifs were associated with a Cdc20 site; Figure 2B, red arrow; *P* = 0.003). Moreover, G4 motifs associated with Pfh1 in WT cells were much more likely to show high Cdc20 association in Pfh1-depleted cells compared to G4 motifs not associated with Pfh1 (*P* = 1.9E-11; Table 3). For example, 43% of the Pfh1-associated G4 motifs were also high Cdc20 binding sites in Pfh1-depleted cells. In contrast, only 11% of the G4 motifs not associated with Pfh1 had high Cdc20 binding in Pfh1-depleted cells. This pattern was also true for G4 motifs and Cdc20 peaks from Pfh1-expressing cells (*P* = 1.5E-17; Table 3). Thus, while G4 motifs as a group were not significantly associated with high Cdc20 in WT cells, those G4 motifs that had high Pfh1 binding were also significantly associated with high Cdc20 binding.

**Table 3:** **G4 motif association with high Pfh1 and Cdc20 occupancy**

**G4 Status**	**Pfh1 Status**	**Number of motifs**	**Number with Cdc20**	**% with Cdc20**	***P*** **-value**
G4 with Pfh1	Expressed	90	36	40%	**1.5E-17**
G4 w/o Pfh1	Expressed	356	14	4%	
G4 with Pfh1	Depleted	90	39	43%	**1.9E-11**
G4 w/o Pfh1	Depleted	356	38	11%	

G4 motifs associated with high Pfh1 occupancy are significantly more likely to be associated with replication fork pausing as indicated by high Cdc20 occupancy in both Pfh1-expressing (*P* = 1.5E-17) (Pfh1 status expressed) and Pfh1-depleted (Pfh1 status depleted) cells (*P* = 1.9E-11). G4, G-quadruplex.

To validate the high Cdc20-binding sites, we performed ChIP-qPCR in WT and Pfh1-depleted cells with the same primer pairs used to confirm Pfh1 association (Figure 2C). At the tRNA gene, and all three G4 motifs, we found significantly higher Cdc20 occupancy in Pfh1-depleted cells compared to WT cells (Figure 2C). Cdc20 occupancy in WT cells and Pfh1-depleted cells did not increase at the three GC-rich regions (Figure 2C).

### DNA damage occurs near G4 motifs

Phosphorylation of histone H2A is one of the earliest responses to a DSB. In *S. pombe*, H2A phosphorylation occurs in an area of approximately 25 kb on either side of a DSB, with the highest peaks around 5 kb from the break site [35]. To determine if fork pausing near G4 motifs resulted in DNA damage, we performed ChIP-seq using antibodies that recognize phosphorylated H2A (γ-H2A) [6,35]. As in the Cdc20 experiments, nmt-Pfh1-GFP cells were grown with or without thiamine for 12 hours and then processed for ChIP-seq and ChIP-qPCR.

We identified 179 γ-H2A peaks in Pfh1-expressing cells and 582 γ-H2A peaks in Pfh1-depleted cells. These peaks were associated with 77 and 177 G4 motifs, respectively (Figure 3A). Even in the presence of Pfh1, G4 motifs were significantly enriched within 5 kb of γ-H2A peaks (Figure 3B, blue arrow and histogram; *P* = 0.021; 77 associations). This association was even stronger in Pfh1-depleted cells, with 177 G4 motifs with high γ-H2A levels (Figure 3B, red arrow and histogram; *P* = 0.014). Thus, G4 motifs were near sites of DNA damage in both Pfh1-expressing and Pfh1-depleted cells, but the number of damage-associated G4 motifs was much higher in the absence of Pfh1.

As for Cdc20 occupancy, we validated the γ-H2A peaks with ChIP-qPCR using anti-γ-H2A antibodies in WT and Pfh1-depleted cells (Figure 3C). Both the tRNA gene and the three G4 motifs had significantly increased γ-H2A levels in Pfh1-depleted cells compared to WT cells (Figure 3C). We did not detect a significant increase for the three investigated GC-rich regions (Figure 3C).

To determine if DNA damage was more likely to occur at individual G4 sites in Pfh1-depleted versus WT cells, we compared the *P*-values for the γ-H2A peaks at a given G4 motif in Pfh1-expressing and Pfh1-depleted cells. We used the same number of ChIP-seq reads from both contexts so that *P*-values from the two conditions could be compared. G4 motifs without an overlapping peak in a given context were assigned a P-value of 1. The mean *P*-value decreased from 0.003 in Pfh1-expressing cells to 1E-14 in Pfh1-depleted cells, and the peak *P*-values in Pfh1-depleted cells were consistently more significant [see Additional file 4; *P* approximately 0, Wilcoxon signed-rank test]. These findings suggest that the probability of DNA damage at a given G4 motif is higher in Pfh1-depleted versus Pfh1-expressing cells.

### In the absence of Pfh1, G4 motifs associated with replication fork stalling are more likely to result in double strand breaks

To combine the observations on G4 motifs, Pfh1 presence, replication fork slowing and DNA damage in Pfh1-depleted cells, we analyzed the association between high Cdc20 occupancy and γ-H2A levels at G4 motifs in Pfh1-expressing and Pfh1-depleted cells. When Pfh1 was expressed, G4 motifs with high Cdc20 occupancy were no more or less likely to be near a site of DNA damage, as marked by γ-H2A, than G4 motifs without Cdc20 (*P* = 0.23, Fisher’s exact test; Table 4). However, when the same test was performed on data from Pfh1-depleted cells, there was a highly significant association between high Cdc20 occupancy at G4 motifs and nearby damage (*P* = 8.7E-6; Table 4). These data suggest that in WT cells Pfh1 prevents breakage of forks that pause at G4 sites.

**Table 4:** **The association between G4 motifs, replication fork pausing and DNA damage is Pfh1 dependent**

**G4 Status**	**Pfh1 Status**	**Number of motifs**	**Number with high** **γH2A**	**% with high** γ**H2A**	***P*** **-value**
G4 motifs with high Cdc20	Expressed	50	11	22%	**0.23**
G4 motifs w/o high Cdc20	Expressed	396	66	17%	
G4 motifs with high Cdc20	Depleted	77	48	62%	**8.7E-6**
G4 motifs w/o high Cdc20	Depleted	369	129	35%	

In Pfh1-depleted cells (Pfh1 status depleted), G4 motifs associated with replication fork pausing (high Cdc20) were much more likely to result in DNA damage (high γH2A occupancy) than G4 motifs that were not associated with fork pausing. Fisher’s exact test was used to calculate *P*-values. G4, G-quadruplex.

### Features of G4 motifs that are Pfh1- and DNA damage-associated

In total, 251 of the 446 G4 motifs in the *S. pombe* genome were associated with Pfh1, fork pausing (Cdc20) and/or DNA damage (γ-H2A). Based on their relationship with Pfh1, we defined three classes of G4 motifs. The first class contained 90 G4 motifs that were Pfh1 associated in WT cells (Class I). The second class consisted of 100 Pfh1-sensitive G4 motifs; that is, these motifs were sites of fork slowing and/or DNA breakage only in Pfh1-depleted cells (Class II). The third class consisted of 106 G4 motifs that were sites of fork slowing and/or DNA damage in both WT and Pfh1-depleted cells (Class III). By definition, there was no overlap between Class II and Class III, but Class I motifs could also be in either Class II or III.

Nearly 40% of Class I motifs were not sites of genome instability in either WT or Pfh1-depleted cells. This finding suggests that these G4 motifs do not form G4 structures, at least during S phase, or that these motifs are resolved by a different helicase in Pfh1-depleted cells. Only eight (9%) of the Pfh1-sensitive (Class II) motifs were Pfh1-associated (Class I) in WT cells, a surprising finding (see Discussion). In contrast, Pfh1 was detected at 40% of Class III sites, but this binding was not sufficient to prevent damage in WT cells. However, pausing, as monitored by levels of Cdc20 occupancy, was higher at 70% of these sites in Pfh1-depleted cells compared to WT cells (*P* = 0.015, Additional file 5); that is, the presence of Pfh1 did facilitate replication at many Class III sites.

We explored genomic features associated with the three categories of G4 motifs to see if any attributes distinguished them from each other and those seen when all G4 motifs were considered together. The 90 Class I G4 motifs lacked the associations seen when considering G4 motifs overall, except for being enriched at meiotic DSB hotspots. The only other significant association for this class was overrepresentation within the 500 most highly transcribed RNA polymerase II genes (36 of the 90 motifs; q <0.008). Remarkably, in almost all (88%) of these cases, the G4 motif was on the transcribed strand of the highly transcribed gene. This strand bias was significantly stronger than the bias observed for G4 motifs overall (87% versus 72%; *P* = 0.0002, Fisher’s exact test). This enrichment was particularly striking given the significant depletion of G4 motifs in ORFs when all G4 motifs were considered (q <0.004; Table 2). Genomic attributes for Class II and III G4 motifs were similar to the patterns observed for G4 motifs overall (Additional file 3).

## Discussion

Although Pif1 family helicases are found in almost all eukaryotes, virtually all *in vivo* evidence for their role at G4 motifs comes from budding yeast. To determine if the deleterious impact of unresolved G4 structures and the positive role of Pif1 family helicases at these structures holds true in other organisms, we used an integrated computational and experimental approach in *S. pombe*, an organism that diverged from *S. cerevisiae* more than a billion years ago. These studies are particularly important because budding yeast is unusual in encoding two Pif1 helicases, ScPif1 and ScRrm3, while most eukaryotes encode only one (reviewed in [4]). The two *S. cerevisiae* helicases, ScPif1 and ScRrm3, have multiple, often conflicting, roles in genome integrity, so it is not clear how findings on ScPif1 and ScRrm3 translate to organisms with a single Pif1 helicase. In addition, by multiple criteria, *S. pombe* chromosomes are more similar to mammalian chromosomes than *S. cerevisiae* chromosomes [36]. Thus, the sole *S. pombe* Pif1 family helicase, Pfh1, is a more apt model for the mammalian enzyme.

### Consistent patterns in the genomic distribution of G4 motifs across three diverse species

The genomic features associated with G4 motifs in *S. pombe* were strikingly similar to those seen in the *S. cerevisiae* and human nuclear genomes [15,26,37], supporting the conservation of G4 motif biology across more than one billion years of evolution. In particular, three functional regions are G4-rich and a fourth is G4-poor in the three organisms. First, promoters of RNA polymerase II transcribed genes in *S. pombe* contained more G4 motifs than expected (Table 2). Likewise in budding yeast and humans, G4 motifs are enriched within 850 bp and 1 kb, respectively, of the transcriptional start site, suggesting a common regulatory function for G4 motifs [17,26]. This correlation also agrees with our finding that *S. pombe* G4 motifs were enriched in NDRs, regions that are found in the majority of promoters [38]. Second, as in budding yeast and humans, the S. pombe rDNA was enriched with G4 motifs [15,26,27,39]. In *S. pombe* and *S. cerevisiae*, they were only present on the non-transcribed strand [15,26]. The fact that the G4 motifs are over-represented in rDNA in evolutionarily diverse organisms argues that this arrangement has functional importance. For example, formation of G4 structures in the non-transcribed strand could facilitate high transcription rates by sequestering the transcribed template to prevent re-annealing to the G4-rich complementary strand. Third, telomeres in yeast, human and many other species contain G4 motifs. *S. pombe* telomeres are no exception; they have the highest density of G4 motifs of any region in the *S. pombe* genome: 4.5 G4 motifs per kb, which is even higher than expected from the high GC content of telomeric DNA. G4 structures form *in vivo* in a cell cycle dependent manner in ciliate and human telomeric DNA [9,10,13], and their presence is proposed to protect ends from nuclease degradation, and/or affect telomerase recruitment [40-42]. The fact that telomeres bear constitutive single-stranded G-tails makes them strong candidates for G4 formation, unless proteins or other structures (for example, t-loops) prevent their formation. The fourth conserved association of G4 motifs in all three organisms is their depletion from ORFs (Table 2) [11,15].

Taken together, these common patterns in the genomic distribution of G4 motifs add to the increasing evidence that G4 structures have regulatory functions that are maintained by selection and that likely counterbalance their negative effects on genome stability [15,16,26,37]. However, in certain contexts, such as ORFs, the price of their negative effects may be too steep, leading to selection against G4 motifs in these regions.

### Pfh1 suppresses G4-induced genomic instability in *S. pombe*

We provide multiple lines of evidence that support the importance of Pfh1 in suppressing G4-induced genomic instability in *S. pombe*. First, 20% of G4 motifs had high Pfh1 occupancy, consistent with the possibility that Pfh1 acts at a subset of G4 motifs *in vivo*. This number is likely an underestimate. For example, only 9% of the Class II Pfh1-sensitive sites had significant Pfh1 binding; yet their dependence on Pfh1 argues that all of these sites may bind Pfh1. We attribute the lack of detectable binding at most Class II sites to the speed with which Pif1 family helicases unwind G4 structures [19], the stringent criteria used to identify binding sites, and technical difficulties detecting DNA helicases by ChIP [18]. In contrast to Class II sites, Pfh1 was detected at 40% of Class III sites. This finding suggests that G4 structures persist longer at Class III than at Class II sites. For example, at Class III G4 motifs, G4 structures may reform after Pfh1 action, leading to repeated cycles of Pfh1 binding and unwinding at the site. This hypothesis could also explain why Class III G4 motifs were sites of replication fork slowing and/or DNA damage even in WT cells. Our data also likely underestimate the fraction of G4 motifs that form G4 structures: other helicases might act at other G4 motifs, while some G4 structures may form only outside of S phase or only in specific growth conditions. Of course, some G4 motifs may rarely or never form G4 structures.

The second result supporting a role for Pfh1 at G4 motifs is that replication pausing at G4 motifs, as monitored by high Cdc20 occupancy, was much higher in Pfh1-depleted than in WT cells (Figure 2). Moreover, G4 motifs that were associated with Pfh1 in WT cells were much more likely to be associated with replication fork pausing than those that were not bound by Pfh1 (Table 3). Third, G4 motifs were significantly associated with DNA damage, as indicated by the presence of γ-H2A. Some G4 motifs were sites of damage even in the presence of Pfh1, but this association and γ-H2A levels were considerably stronger in its absence (Figure 3; Additional file 4). Moreover, when Pfh1 was depleted, G4 motifs with replication fork pausing (high Cdc20) were much more likely to result in DNA damage than G4 motifs without pausing (62% versus 35%; Table 1).

As in budding yeast and humans, G4 motifs in *S. pombe* were underrepresented within ORFs (Table 2). However, when *S. pombe* G4 motifs did occur in ORFs, they were enriched on the transcribed strand (72%; *P* <1E-12). This enrichment was particularly marked for the Pfh1-associated G4 motifs (87%). Furthermore, this enrichment was especially high among the top 500 most highly transcribed RNA polymerase II genes (94%; *P* <0.001; Additional file 6). Although a recent report demonstrated that highly expressed genes may be biased for high ChIP-seq signals [43], fork slowing in highly expressed *S. pombe* genes and its increase in Pfh1-depleted cells, are detected by two-dimensional gels as well as by ChIP [6]. The G4 motifs in ORFs tended to fall within the first half of the ORF (62%), but G4 motifs were observed near the ends of genes as well [see Additional file 7]. A similar strand bias was seen when only considering G4 motifs that overlapped 5′ and 3′ UTRs, 74 % (35/47) and 77% (37/48), respectively, were found on the transcribed strand. A G4 motif on the transcribed strand is expected to inhibit RNA polymerase progression. One possibility is that this class of G4 motifs regulates transcription elongation: transcription could start but then pause at the G4 motif until the regulated recruitment of Pfh1 allows G4 unwinding and resumption of transcription. This type of regulation is particularly appealing in multi-cellular organisms where developmentally regulated genes are often controlled by activation of a paused RNA polymerase [44]. Alternatively, Pfh1 bound to G4 motifs within highly transcribed genes might facilitate RNA removal and inhibit R-loop formation. This speculation is based on the unusual property of budding yeast Pif1, which has higher unwinding activity on RNA/DNA compared to DNA/DNA hybrids [45].

## Conclusions

The evolutionary conservation of G4 motif enrichment in promoters, rDNA and telomeres in two distantly related yeasts and humans argues that they have functions in each of these regions. Their depletion from the protein-coding ORFs in the three organisms, as well as their association with fork stalling and DNA breakage in the two yeasts, demonstrates that their positive roles come with a negative impact on genome integrity. These negative effects are mitigated in both *S. cerevisiae* and *S. pombe* by the action of Pif1 family helicases. We propose that processing of G4 structures by Pif1 family helicases is a common and evolutionarily conserved mechanism.

## Methods

### ChIP and preparation of libraries for DNA sequencing

All yeast strains used in this study are listed in Additional file 8. ChIP experiments were performed as described previously [6]. Briefly, cells were cross-linked in 1% formaldehyde at 25°C for five minutes. The chromatin was sheared to an average of approximately 300 bps length with a Covaris E220 system and immunoprecipitated with anti-Myc antibody (Clontech Laboraties, Mountain View, California, USA Cat. nr 631206), γ-H2A antibody (a kind gift from C. Redon) or anti-HA antibody (Santa Cruz biotechnologies, Dallas, Texas, USA Cat nr. sc.7392x). Both input and immunoprecipitated DNA were purified and quantified by real-time PCR using primers for *ade6* and *STE*. A sample of 20 ng DNA was used to prepare a sequencing library with the TruSeq DNA sample preparation kit v2 (Illumina, San Diego, California, USA Cat nr. FC-121-2001) as described by the manufacturer. The libraries were sequenced with the Illumina HiSeq 2000 sequencing platform at Princeton University (Princeton, NJ, USA). Two biological replicates were sequenced for each experiment.

### ChIP combined with quantitative PCR

ChIP experiments were performed as described above. Both input and immunoprecipitated DNA were purified and quantified by quantitative PCR (Roche Diagnostics, Indianapolis, Indiana, USA Lightcycler® 96 instrument) with either primer pairs for tRNA glu.05, GC_1, GC_2, GC_3, G4_1, G4_2, or G4_3 [see Additional file 9 for primer sequences]. For γ-H2A ChIP-qPCR, the primer pairs were designed within a 5 kb region of the tRNA gene, the GC-rich region, and the G4-motifs [see Additional file 9]. For strains expressing Pfh1-13Myc, an untagged strain was used as a control (YSP3; Additional file 8). The IP/input ratio was calculated by dividing immunoprecipitated DNA by input DNA. The data presented are an average of three independent cultures.

### Sequence analysis and peak calling

The 101 bp single-end sequencing reads were analyzed using tools available in the Galaxy platform [46]. Reads were mapped to the *S. pombe* genome (Pombase_09052011) [47] using Bowtie [48], permitting two mismatches in the seed (seed length 28 bp). Reads were mapped to the genome for both input and ChIP samples. Only uniquely mapped reads were included in the alignments. Peaks were identified with MACS1.4 [49] using the following settings: bandwidth of 300 (DNA shearing size), *P*-value cutoff of 10^-5^ and tag size of 101. Input DNA was used as the control. The average peak size was 1,838 ± 983 bp (± standard deviation) for the 621 Pfh1-associated regions, 1,526 ± 717 bp for the 485 high Cdc20-associated regions in Pfh1-expressing cells and 1,666 ± 860 bp for the 517 high Cdc20-associated regions in Pfh1-depleted cells. The 179 high γ-H2A peaks in Pfh1-expressing cells were 1,486 ± 742 bp, and the 582 γ-H2A peaks in Pfh1-depleted cells were 1,974 ± 1071 bp. All sequence data are deposited with the GEO accession number GSE59178.

### G4 motif identification

The genome sequences and alignments for *S. pombe*, *S. octosporus*, *S. cryophilus* and *S. japonicus* were downloaded from the supporting web site for Rhind *et al*. [22] on May 3, 2012. We identified the location of all DNA sequences in the nuclear and mt genomes with the potential to form G4 structures (‘G4 motifs’) using a previously described regular expression search program with a minimum G-island length of 3 and a maximum loop length of 25 bp [15]. Because the current *S. pombe* genome assembly does not include telomeres, we also scanned the sequence of DNA in a recently sequenced telomere library (Webb and VAZ, submitted). Using alignments of the nuclear genomes of the four fission yeasts, we analyzed G4 motif evolutionary conservation. In this analysis, any G4 motif for which the aligned portion of another genome contained a G4 motif was considered conserved between the two species.

### Analysis of G4 motif associations with genomic features

To explore potential functions for G4 motifs, we compared their genomic distribution in *S. pombe* with known functional genomic regions. We considered the following genome annotations: ORFs, dubious genes, essential genes, highly expressed genes [22], origins of replication from OriDB [50], telomeres (Webb and VAZ, submitted), LTRs, 5S rRNA, tRNAs, centromeres, NDRs [see Additional file 10] [38] called using Podbat [51], rDNA repeats and meiotic DSB hot spots [52]. Unless otherwise noted, these annotations were taken from PomBase [47]. The highly transcribed genes were determined from the RNA-seq data collected by [22]. We took the 500 genes with the highest expression in fragments per kilobase per million reads (FPKM) from their combined dataset, which summarized expression patterns from cells in log phase, glucose depletion, early stationary phase, and heat shock [see Additional file 6]. Results were similar when we considered only the 100 most highly expressed genes.

Regions that overlapped a G4 motif were considered to be associated with the G4. We also computed the association of G4 motifs with the Pfh1, Cdc20 and γ-H2A peaks determined here by ChIP-seq. For the γ-H2A analysis, a 5 kb window on both sides was used to determine G4 motif associations, as DNA damage results in maximal phosphorylation in a roughly 5 kb region on either side of the break [35]. For Pfh1 and Cdc20 peaks, a window of 300 bp, the DNA shearing size, was used, but our main results held with windows of 0 and 500 bp.

To evaluate the significance of observed associations between G4 motifs and genomic annotations, we performed simulations to obtain an empirical *P*-value. The number of associations expected at random if there were no functional relationship between two sets of genomic regions (for example, G4 motifs and essential genes) depends on many factors, including their lengths, distribution across the chromosomes, and nucleotide contents. To account for these factors, we generated 1,000 random ‘control’ sets of genomic regions. Each of these sets consisted of 446 individual genomic regions—one for each of the observed G4 motifs. Each of these individual regions was randomly placed on the genome with three constraints. First, it had to be on the same chromosome as its corresponding G4 motif; second, it had to have the same length as the G4 motif; and third, it had to have the same GC content as the G4 motif. As a result, each of the 1,000 random control region sets had the same length, chromosome and GC content distribution as the actual G4 motifs. To obtain a *P*-value for an observed association between the G4 motifs and a genomic annotation of interest, we compared it to the number of associations for each of the 1,000 control region sets with the same annotation. The *P*-value was the number of the random control sets in which a more extreme association was observed. When indicated, we also considered control region sets that were not constrained to match the GC content of the G4 motifs. To account for the testing of multiple hypotheses in this analysis, we computed q-values from the *P*-values using the *qvalue* program [53]. The q-value for a test is the FDR that results if that test is called significant. All associations with q less than 0.05 were considered significant.

## Additional files

Additional file 1:
**Location and sequence of G4 motifs in the genomes of four fission yeast species.**
Additional file 2:
**List of** ***S. pombe*** **telomere sequences.**
Additional file 3:
**Analysis of association of genomic features for several classes of G4 motifs.**
Additional file 4:
**Comparison of G4 motif** **γ-H2A peak** ***P*** **-values in Pfh1-depleted cells and Pfh1-expressing cells.** Each circle represents a G4 motif. G4 motifs for which the maximum nearby γ-H2A peak *P*-value was more extreme in Pfh1-depleted cells are colored red; G4 motifs with the more extreme γ-H2A peak *P*-value in Pfh1-expressing cells are colored blue. The γ-H2A peak *P*-values observed in Pfh1-depleted cells are significantly more extreme than those in Pfh1-expressing cells (*P* ≈ 0, Wilcoxon signed-rank test). G4 motifs that did not overlap γ-H2A peaks were plotted at zero. We obtained similar results when only considering G4 motifs associated with peaks in both contexts (*P* = 2.9E-6). The same number of ChIP-seq reads were used in both contexts to identify γ-H2A peaks and *p*-values. Additional file 5:
**G4 motifs associated with Pfh1 and Pfh1-independent DNA damage.** G4 motifs associated with Pfh1 (class I) and Pfh1-independent DNA damage (class III) have more extreme Cdc20 occupancy peak *P*-values in Pfh1-depleted cells than Pfh1-expressing cells (*P* = 0.015, Wilcoxon signed-rank test). Each circle represents a G4 motif. G4 motifs for which the maximum nearby Cdc20 peak *P*-value was more extreme in Pfh1-depleted cells are colored red; G4 motifs with more extreme Cdc20 peak *P*-value in Pfh1-expressing cells are colored blue. The same numbers of ChIP-seq reads were used in both contexts to identify Cdc20 peaks and *P*-values. Additional file 6:
**List of the 500 highest transcribed genes.**
Additional file 7:
**Pfh1-associated G4 motifs are enriched on the transcribed strand.** (A) Pfh1-associated G4 motifs (class I) are significantly enriched on the transcribed strand of genes. They also show a slight bias to occur in the first half of the ORF, but they are observed near the ends of genes as well. The histogram gives the number of G4 motifs found at a given fraction of the total length of the gene across all class I G4 motifs in ORFs. (B) Similar patterns are observed for Pfh1-associated G4 motifs found in highly transcribed genes. Additional file 8:
**List of yeast strains used in this study.**
Additional file 9:
**List of primer pairs used for ChIP-qPCR.**
Additional file 10:
**List of the genomic location, the average signal intensity and length of NDRs.**
