# Supplementary figures and images for: The essential Schizosaccharomyces pombe Pfh1 DNA helicase promotes fork movement past G-quadruplex motifs to prevent DNA damage
Source: BMC Biol. 2014 Dec 4;12:101. doi: 10.1186/s12915-014-0101-5 (PMC4275981; doi:10.1186/s12915-014-0101-5)

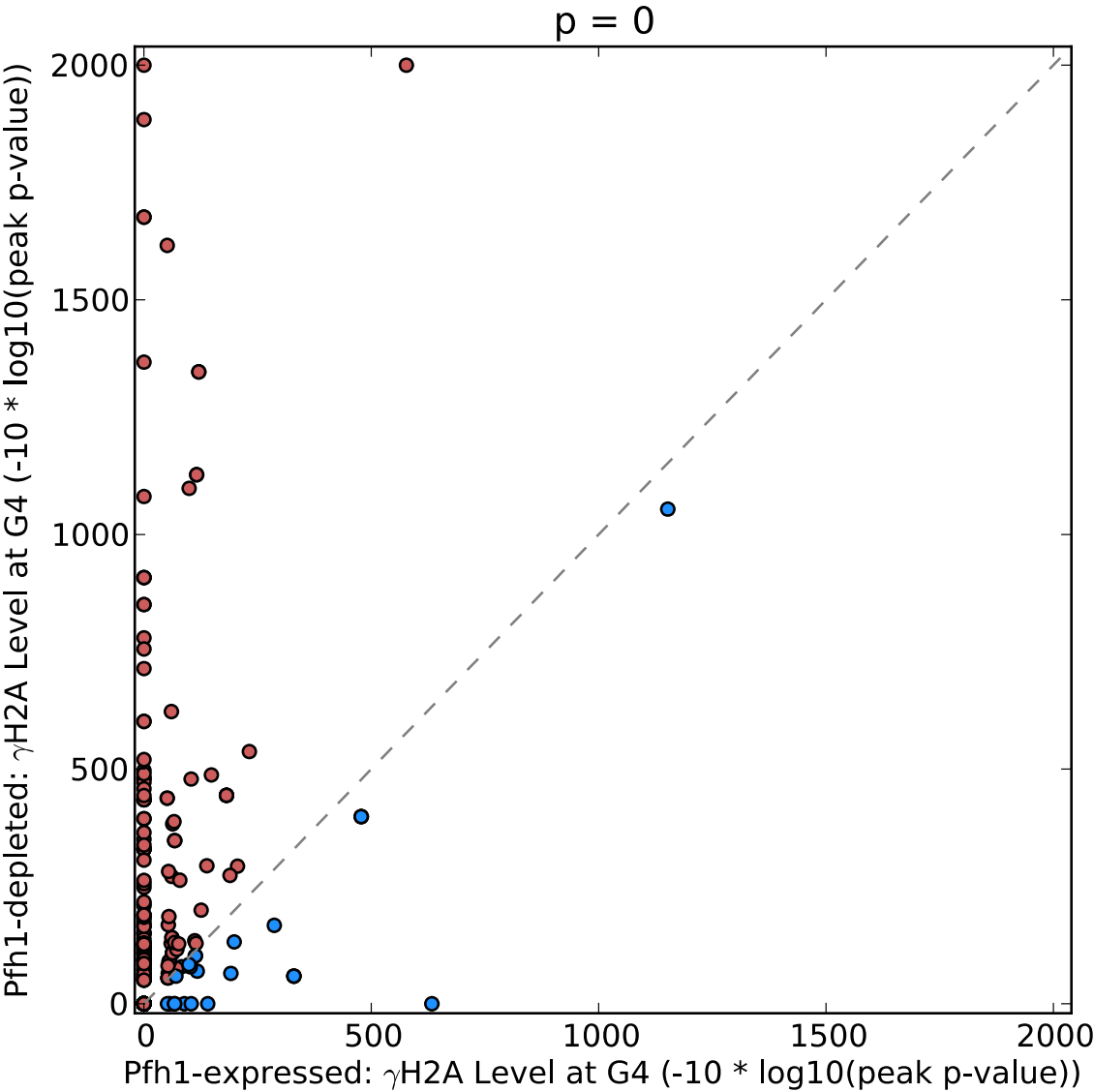

Supplement: Additional file 4: — Comparison of G4 motif γ-H2A peak P -values in Pfh1-depleted cells and Pfh1-expressing cells. Each circle represents a G4 motif. G4 motifs for which the maximum nearby γ-H2A peak P-value was more extreme in Pfh1-depleted cells are colored red; G4 motifs with the more extreme γ-H2A peak P-value in Pfh1-expressing cells are colored blue. The γ-H2A peak P-values observed in Pfh1-depleted cells are significantly more extreme than those in Pfh1-expressing cells (P ≈ 0, Wilcoxon signed-rank test). G4 motifs that did not overlap γ-H2A peaks were plotted at zero. We obtained similar results when only considering G4 motifs associated with peaks in both contexts (P = 2.9E-6). The same number of ChIP-seq reads were used in both contexts to identify γ-H2A peaks and p-values. [file 12915_2014_101_MOESM4_ESM.pdf]

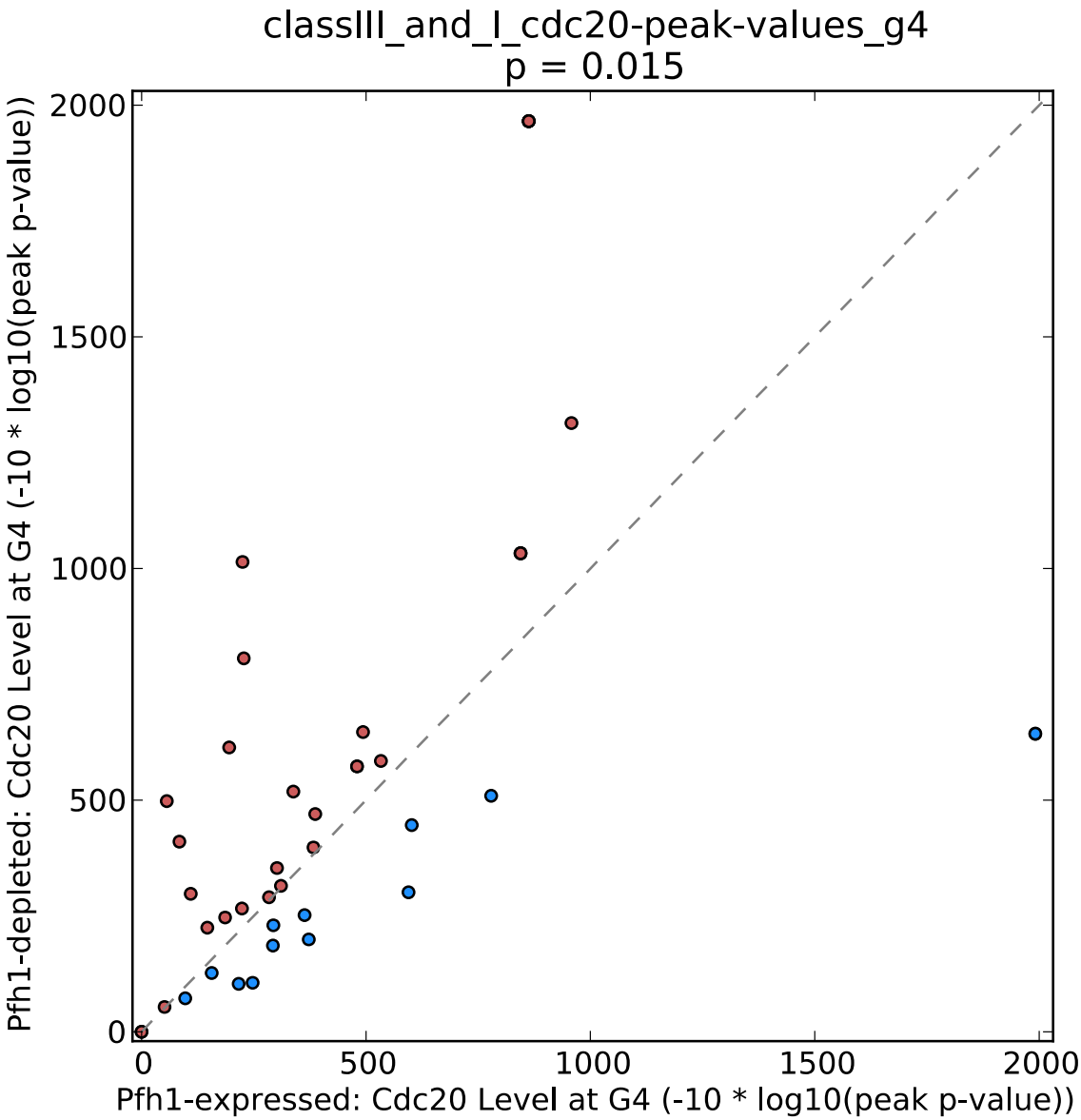

Supplement: Additional file 5: — G4 motifs associated with Pfh1 and Pfh1-independent DNA damage. G4 motifs associated with Pfh1 (class I) and Pfh1-independent DNA damage (class III) have more extreme Cdc20 occupancy peak P-values in Pfh1-depleted cells than Pfh1-expressing cells (P = 0.015, Wilcoxon signed-rank test). Each circle represents a G4 motif. G4 motifs for which the maximum nearby Cdc20 peak P-value was more extreme in Pfh1-depleted cells are colored red; G4 motifs with more extreme Cdc20 peak P-value in Pfh1-expressing cells are colored blue. The same numbers of ChIP-seq reads were used in both contexts to identify Cdc20 peaks and P-values. [file 12915_2014_101_MOESM5_ESM.pdf]

Additional file 7

A

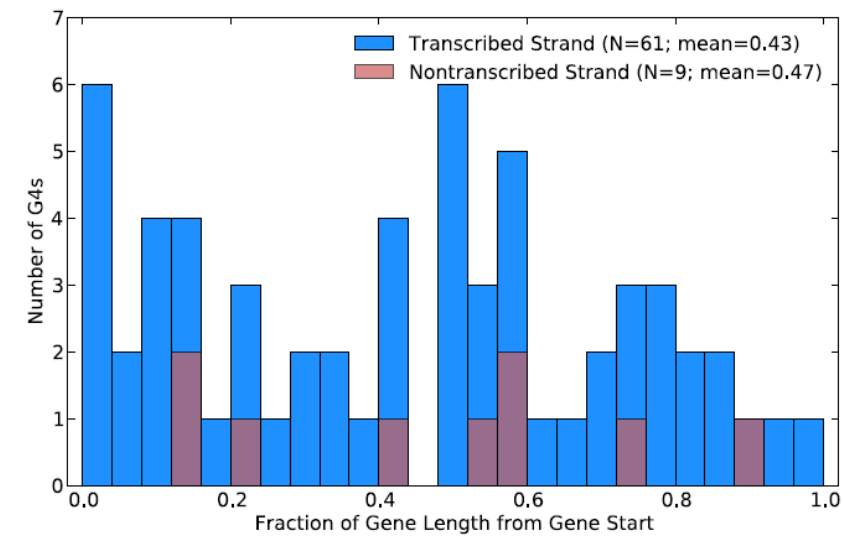

B

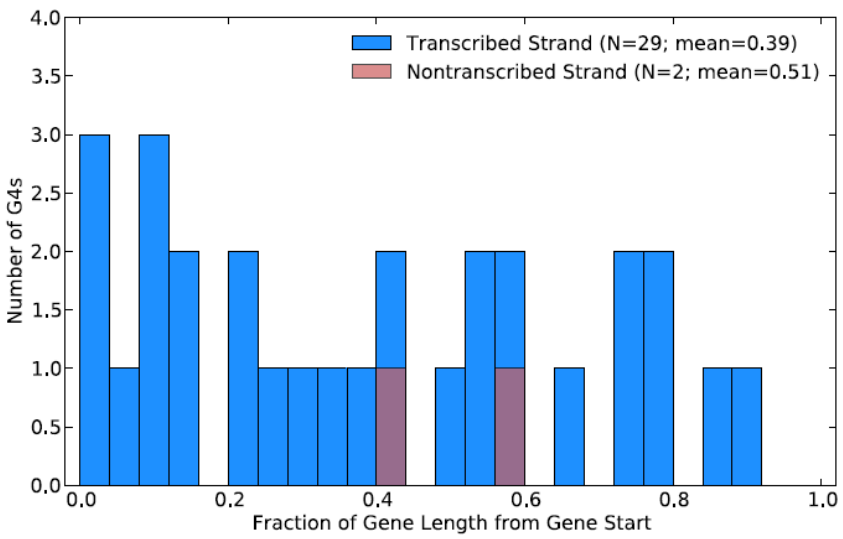

Supplement: Additional file 7: — Pfh1-associated G4 motifs are enriched on the transcribed strand. (A) Pfh1-associated G4 motifs (class I) are significantly enriched on the transcribed strand of genes. They also show a slight bias to occur in the first half of the ORF, but they are observed near the ends of genes as well. The histogram gives the number of G4 motifs found at a given fraction of the total length of the gene across all class I G4 motifs in ORFs. (B) Similar patterns are observed for Pfh1-associated G4 motifs found in highly transcribed genes. [file 12915_2014_101_MOESM7_ESM.pdf]
