# Supplementary material for: The essential Schizosaccharomyces pombe Pfh1 DNA helicase promotes fork movement past G-quadruplex motifs to prevent DNA damage
Source: BMC Biol. 2014 Dec 4;12:101. doi: 10.1186/s12915-014-0101-5 (PMC4275981; doi:10.1186/s12915-014-0101-5)
Supplement: Additional file 8: — List of yeast strains used in this study. [file 12915_2014_101_MOESM8_ESM.docx]

Additional file 8. Yeast strains used in this study

| Strain | Genotype | Source |
| --- | --- | --- |
| YNS29 | h^-^ *ade6-210 leu1-32::pJk148-pfh1-13Myc-kanmx6 ura4-D18 his3-D1* | (Sabouri et al. 2012) |
| YSA60 | h^+^ *ade6-M21x leu1-32 ura4-D18 his3-D1 telo-his3+ pfh1::ura4^+^-nmt-pfh1-GFP* | (Pinter et al. 2008)^a^ |
| YNS119 | h^-^ *cdc20+::cdc20-3HA-kanmx6 pfh1::ura4+-nmt-pfh1-GFP ura4-D18 leu1-32 his3-D1* | This study |
| YSP3 | h^-^ *leu1-32 his3-D1 ura4-D18 ade6-M210* | (Pinter et al. 2008) |

*derivative of YSP383 (Pinter at al. 2008)
