# Supplementary material for: The essential Schizosaccharomyces pombe Pfh1 DNA helicase promotes fork movement past G-quadruplex motifs to prevent DNA damage
Source: BMC Biol. 2014 Dec 4;12:101. doi: 10.1186/s12915-014-0101-5 (PMC4275981; doi:10.1186/s12915-014-0101-5)
Supplement: Additional file 9: — List of primer pairs used for ChIP-qPCR. [file 12915_2014_101_MOESM9_ESM.docx]

Additional file 10. Primer sequences used for quantitative PCR

| **Site** | **Sequence 5’-3’** |  | **Strand** | **Location of site** | **ChIP** |
| --- | --- | --- | --- | --- | --- |
| G4_1 | ATCATCATCACCGTCCGTCG | | Forward | chrII_524370 | Pfh1/Cdc20 |
| G4_1 | ACATTCGCAAACCCTGACCT | | Reverse | chrII_524370 | Pfh1/Cdc20 |
| G4_1 | GCTCTCTCTGCATCATCGCT | | Forward | chrII_524370 | γ-H2A |
| G4_1 | ATATGCCGGTCGTTTGGGTT | | Reverse | chrII_524370 | γ-H2A |
| G4_2 | AACCGTTTGTGTCATTGGCG | | Forward | chrII_2111669 | Pfh1/Cdc20 |
| G4_2 | CCCTGCCTTCCCAAACTCAT | | Reverse | chrII_2111669 | Pfh1/Cdc20 |
| G4_2 | AACCGTTTGTGTCATTGGCG | | Forward | chrII_2111669 | γ-H2A |
| G4_2 | CCCTGCCTTCCCAAACTCAT | | Reverse | chrII_2111669 | γ-H2A |
| G4_3 | ATGCAACGTTTCACCGCTTC | | Forward | chrII_1269935 | Pfh1/Cdc20 |
| G4_3 | AGCGACCATAAGGAACGTCG | | Reverse | chrII_1269935 | Pfh1/Cdc20 |
| G4_3 | CGTTTCTCGCTGCTTGCTTT | | Forward | chrII_1269935 | γ-H2A |
| G4_3 | GCTGACTTTTTACCCCCGGA | | Reverse | chrII_1269935 | γ-H2A |
| GC_1 | CTGCCTCGCCATAGTCTTGT | | Forward | chrI_218950 | Pfh1/Cdc20 |
| GC_1 | TGGGGAGTGCTATGAACACG | | Reverse | chrI_218950 | Pfh1/Cdc20 |
| GC_1 | ACGTGTTTTGTTTACGGCAGA | | Forward | chrI_218950 | γ-H2A |
| GC_1 | TGAAATTTCCAGCGACTGCT | | Reverse | chrI_218950 | γ-H2A |
| GC_2 | TTGACCAAAAGCATGGCTGC | | Forward | chrII_1974700 | Pfh1/Cdc20 |
| GC_2 | GCAACTTCTCGTCTTTGGCG | | Reverse | chrII_1974700 | Pfh1/Cdc20 |
| GC_2 | TCTTTCATGCCAGCTTCGGT | | Forward | chrII_1974700 | γ-H2A |
| GC_2 | GCTGGTCTTGTTGGTACGGA | | Reverse | chrII_1974700 | γ-H2A |
| GC_3 | CACGACAACGGCCCAAAAAT | | Forward | chrII_2227750 | Pfh1/Cdc20 |
| GC_3 | GGACAAAACAGCGCTTGGAA | | Reverse | chrII_2227750 | Pfh1/Cdc20 |
| GC_3 | TCCATGCTTGGTTCTGCTGT | | Forward | chrII_2227750 | γ-H2A |
| GC_3 | TTCCGGTCCAGCTCAGAAAA | | Reverse | chrII_2227750 | γ-H2A |
| tRNA glu.05 | CAAAGATTGGGATGCTATGACAC | | Forward | chrII_820491 | Pfh1/Cdc20 |
| tRNA glu.05 | TGCGTACAAGACTTGGATGAG | | Reverse | chrII_820491 | Pfh1/Cdc20 |
| tRNA glu.05 | TAGCAGATGAATACCGTGAGTC | | Forward | chrII_820491 | γ-H2A |
| tRNA glu.05 | CATTAAGGAACCATTAGATTAAACCG | | Reverse | chrII_820491 | γ-H2A |
